# Shyness discriminates between children with 22q11.2 deletion syndrome and Williams syndrome and predicts emergence of psychosis in 22q11.2 deletion syndrome

**DOI:** 10.1186/1866-1955-6-3

**Published:** 2014-02-11

**Authors:** Yael Schonherz, Maayan Davidov, Ariel Knafo, Hadas Zilkha, Gal Shoval, Gil Zalsman, Amos Frisch, Abraham Weizman, Doron Gothelf

**Affiliations:** 1 The Behavioral Neurogenetics Center, The Edmond and Lily Safra Children’s Hospital, Sheba Medical Center, Tel Hashomer 52621, Israel; 2 School of Social Work and Social Welfare, The Hebrew University of Jerusalem, Mount Scopus, Jerusalem 91905, Israel; 3 Department of Psychology, The Hebrew University of Jerusalem, Mount Scopus, Jerusalem 91905, Israel; 4 Sackler Faculty of Medicine, Tel Aviv University, Tel Aviv 69978, Israel; 5 Child and Adolescent Psychiatry Division, Geha Mental Health Center, Petah Tikva 49202, Israel; 6 Felsenstein Medical Research Center, Petah Tikva 49202, Israel

**Keywords:** Psychosis, Shyness prediction, Temperament, Velocardiofacial syndrome, Williams syndrome

## Abstract

**Background:**

22q11.2 deletion syndrome (22q11.2DS) is a common neurogenetic syndrome associated with high rates of psychosis. The aims of the present study were to identify the unique temperament traits that characterize children with 22q11.2DS compared to children with Williams syndrome (WS) and typically developing (TD) controls, and to examine temperamental predictors of the emergence of psychosis in 22q11.2DS.

**Methods:**

The temperament of 55 children with 22q11.2DS, 36 with WS, and 280 TD children was assessed using the Emotionality, Activity, Sociability (EAS) Temperament Survey, Parental Ratings. The presence of a psychotic disorder was evaluated in 49 children and adolescents with 22q11.2DS at baseline and again 5.43 ± 2.23 years after baseline temperament assessment.

**Results:**

Children with 22q11.2DS scored higher on Shyness compared to WS and TD controls. Children with 22q11.2DS and WS scored higher on Emotionality and lower on Activity compared to TD controls. Shyness was more severe in older compared to younger children with 22q11.2DS. Baseline Shyness scores significantly predicted the later emergence of a psychotic disorder at follow-up, in children with 22q11.2DS.

**Conclusions:**

Our results suggest that shyness is an early marker associated with the later emergence of psychosis in 22q11.2DS.

## Background

Genetic syndromes with well-defined etiologies provide an excellent opportunity for examining the contributions of genetic defects to unique behaviors. The 22q11.2 deletion syndrome (22q11.2DS) and Williams syndrome (WS) are known neurogenetic autosomal dominant syndromes, both caused by a microdeletion, 22q11.2DS in the long arm of chromosome 22 [1] and WS in the long arm of chromosome 7 (7q11.23) [2]. Both 22q11.2DS and WS are characterized by physical and psychiatric comorbidities and cognitive deficits [3,4]. The two syndromes are associated with high rates of psychiatric disorders including attention deficit/hyperactivity disorder (ADHD), oppositional defiant disorder (ODD), and phobias. Social anxiety disorder is more prevalent in 22q11.2DS while specific phobia is more common in WS [5-7]. Up to one-third of individuals with 22q11.2DS develop schizophrenia-like psychotic disorders during adolescence and early adulthood, making 22q11.2DS the most commonly known genetic syndrome associated with schizophrenia [8,9].

Research evidence indicates that individuals with developmental disabilities and neurogenetic syndromes have many common characteristics, including increased rates of psychiatric disorders (e.g., ADHD and anxiety disorders), cognitive deficits (e.g., reward circuitry and executive dysfunctions), and behavioral features (e.g., repetitive behaviors) [10-12]. Individuals with 22q11.2DS and WS also share common medical comorbidities (e.g., calcium dysregulation and cardiovascular anomalies) and neuroanatomical aberrations (e.g., pronounced decreased volumes in parieto-occipital regions) [1,3,5,13,14]. Although quite a few studies have examined the behavioral phenotypes of 22q11.2DS and WS, only a few studies have directly compared the behavioral phenotypes of these two syndromes. Comparing behavioral phenotypes, such as temperament, between neurogenetic syndromes is important for identifying which features are common to both syndromes (suggesting non-specific effects of coping with a developmental disability) and which features are specific to each syndrome (suggesting a more specific genetic cause).

Despite the distinctive personality traits of individuals with 22q11.2DS and WS, only a few empirical studies have addressed the temperamental characteristics of these syndromes. A study that investigated the temperament of elementary school children with 22q11.2DS and WS [15] found that compared to typically developing (TD) children, both 22q11.2DS and WS children were less emotionally stable, less conscientious, and more irritable and dependent. Another study found that in comparison to their siblings and to community controls, children with 22q11.2DS were rated by their parents as being less regular in their daily habits, less able to focus/sustain attention, less cheerful/pleasant, less likely to stay with an activity for a long time, and less able to respond flexibly to changes [16]. Compared to TD children and to individuals with other developmental disabilities, individuals with WS were rated as significantly more approaching, people-oriented, intensive, empathic, gregarious, distractible, susceptible to negative mood, and lower on persistence and threshold of excitability [17,18].

In addition to comparing and differentiating the two syndromes, examining the temperamental features of 22q11.2DS could also help distinguish between different developmental trajectories within this group. Specifically, because psychosis is so common in 22q11.2DS, identifying early risk factors for the subsequent emergence of psychotic disorders in this at-risk population is of great interest. Certain temperamental characteristics may serve as early markers of increased risk for the later development of psychosis in this population.

Therefore, the present study had two main objectives: i) to identify the unique temperament that characterizes children with 22q11.2DS and distinguish them from children with WS and TD controls. We hypothesized that shyness and sociability would distinguish between 22q11.2DS (tending to be shy and less social) and WS (who are extremely outgoing and thus lower on shyness and high on sociability). Further, we predicted that both 22q11.2DS and WS would exhibit more negative emotionality and more activity compared to TD controls given the increased rate of ODD and ADHD in both patient groups [6,7]. ii) To identify temperamental predictors of the subsequent development of psychosis in 22q11.2DS. Based on findings of previous studies on predictors of psychosis in 22q11.2DS [8] and on schizophrenia studies [19-21], we hypothesized that shyness in childhood would be a risk factor for later development of psychosis in 22q11.2DS.

## Methods

### Participants

The study included Jewish individuals with 22q11.2DS, WS, and TD children. Subjects with 22q11.2DS and WS were recruited from the Behavioral Neurogenetic Center of a large tertiary referral center in Israel that coordinates research and treatments of individuals with 22q11.2DS and WS from all over Israel, referred from genetic clinics and parents’ associations. The diagnosis of 22q11.2DS and WS was confirmed in all subjects using fluorescent in situ hybridization test. TD controls were recruited from preschool and elementary schools in the Jerusalem area for studies on child development. The socioeconomic status of both the clinical and control subjects was similar to the range in the general Jewish population in Israel.

The study protocol was approved by the Institutional Review Board of Rabin Medical Center and The Ethics Committee of The Hebrew University of Jerusalem with informed consent obtained from all participants and/or their parents or guardians.

### Measures

The baseline assessment was conducted between 2001 and 2006 (Time 1), and included temperament, psychiatric, and cognitive assessments. A follow-up psychiatric assessment was conducted between 2004 and 2010 for the 22q11.2DS group only (Time 2).

IQ was measured in the 22q11.2DS and WS groups using the age-appropriate versions of the Wechsler Intelligence test [6]. For the psychiatric assessment, individuals and their parents were interviewed by skilled clinicians using the Hebrew version of the Schedule for Affective Disorders and Schizophrenia for School-Aged Children, Present and Lifetime, and adult patients were interviewed using the Structured Clinical Interview for Axis I DSM-IV, as previously described [6].

Temperament was rated by parents, using the Emotionality, Activity, Sociability (EAS) Temperament Survey for Children: Parental Ratings of Buss and Plomin [22]. The EAS was initially designed for children aged 1 to 9 years [22], but its use has been successfully extended to adolescents (11 to 16 years old) [23] and adults [24]. This questionnaire includes 4 subscales – Emotionality, Activity, Sociability, and Shyness, with five items each (including 6 reverse-scored items). The parent rates each item on a scale ranging from 1 = “not characteristic of the child” to 5 = “very characteristic of the child”. A mean score is calculated for each subscale. Thus, scores range from 1 to 5 for each subscale. The four subscales or dimensions of temperament are: *Emotionality:* the tendency to become upset easily and intensely (e.g., “Child gets upset easily”); *Activity*: the tendency to be energetic or vigorous (e.g., “Child is always on the go”); *Sociability*: the tendency to prefer being with others over being alone and to seek social interaction (e.g., “Child likes to be with people”); *Shyness:* the tendency to be fearful, wary, or withdrawn in novel social situations (e.g., “It takes child a long time to warm up to strangers”) [25,26].

The EAS subscales have been shown to possess good psychometric qualities. Their reliability is reflected by both internal consistency and inter-rater agreement [27-29]. Validity evidence includes, for example, the finding that EAS-identified temperament profiles of emotionality and low sociability in young children predict the subsequent development of anxiety, depression, and attention problems later in life [23,30]. Moreover, the EAS scales have been used in numerous clinical studies with children and adolescents and successfully distinguish children with various anxiety disorders and depression and children with developmental disabilities (e.g., Costello syndrome) from normal controls [23,31,32].

### Procedure and data analysis

To identify the unique temperament that characterizes children with 22q11.2DS and WS we compared children aged 2.4 to 12.6 years with 22q11.2DS (n = 55), WS (n = 36), and TD controls (n = 280). The three groups were matched for age (F(2,368) = 1.3, *P* = 0.3) and gender (*P* = 0.2) (Table 1). Differences in temperament between the groups were examined using analysis of covariance (ANCOVA) with Tukey Post-Hoc comparisons. EAS subscale scores were normally distributed and were the dependent variables. In all ANCOVAs, group and sex were used as fixed factors and age as a covariate. IQ score was not entered as a covariate as it was not measured in the TD controls, but links with IQ were assessed in the 22q11.2DS and WS group using correlations and follow-up analyses of covariance. To further examine the association between age and Shyness in 22q11.2DS, we also compared the Shyness scores of 22q11.2DS individuals who were below vs. above the sample’s median age, using an unpaired *t*-test (median age for the 22q11.2DS sample was 6.8 years).

**Table 1:** Age and gender distribution of study samples

**Sample**	**n**	**Age range**	**Mean**	**Median**	**Males/Females**
**(A) 22q11.2DS**	55	3.5–12.5	7.6 (2.9)	6.8	32/23
**WS**	36	2.4–12.2	7.2 (3.0)	7.3	14/22
**TD**	280	3.0–12.6	7.0 (2.9)	7.0	149/131
**(B) 22q11.2DS**					
Baseline	49	5.5–20.9	10.9 (4.9)	11.0	27/22
Follow-up		7.7–26.4	16.3 (5.2)	15.9	

(A) The between group comparisons sample. (B) The 22q11.2 deletion syndrome longitudinal sample.

To identify temperamental predictors of psychosis within 22q11.2DS, we included all individuals with 22q11.2DS who fulfilled the following criteria: i) were below the age of 21 years at baseline assessment; ii) underwent a baseline temperament and psychiatric assessment; and iii) underwent a follow-up psychiatric assessment. Of the 55 individuals with 22q11.2DS, 33 fulfilled the above mentioned criteria and were included in the longitudinal prediction analysis. An additional 16 adolescents and young adults, who were below the age of 21 years at baseline, were also included in the prediction analysis. They were not part of the 22q11.2DS vs. WS and TD comparisons, as they were older than the WS and TD samples. Altogether, 49 individuals (27 males and 22 females) with 22q11.2DS with baseline temperament evaluations and baseline and follow-up psychiatric assessments, were included in the prediction analysis. At baseline, individuals were 10.9 ± 4.9 years and the mean time interval between assessments was 5.4 ± 2.2 years (Table 1). To examine whether temperament predicted emergence of psychosis in 22q11.2DS, a logistic regression was performed with the presence/absence of a psychotic disorder at follow-up entered as the outcome variable. The predicting variables were gender, age, full scale IQ at baseline assessment, and the baseline EAS subscale scores. Furthermore, baseline Shyness scores of 22q11.2DS individuals who developed psychosis were compared to those who did not develop psychosis using an unpaired *t*-test.

## Results

### Temperamental characteristics of children with 22q11.2DS and WS

As hypothesized, we found a significant main effect of group status on Shyness (F(2,368) = 14.6, *P* <0.0001). Scheffe’s post-hoc comparisons showed that children with 22q11.2DS were the shyest, while children with WS were the least shy, compared to the TD controls (Table 2). As hypothesized, we also found a significant main effect of group on Emotionality (F(2,368) = 15.4, *P* <0.0001). Children in both syndrome groups were rated as significantly more emotional than the TD control group (Table 2). A significant group main effect was also found for Activity (F(2,368) = 26.7, *P* <0.0001). Contrary to our hypothesis, children from the TD control group were rated as significantly more active compared to both children with WS and 22q11.2DS (Table 2). There was no significant main effect of group on the Sociability subscale (Table 2).

**Table 2:** Comparison of demographic characteristics and the Emotionality, Activity, Sociability (EAS) Temperament subscale scores between 22q11.2 deletion syndrome (22q11.2DS), Williams syndrome (WS) and typically developing (TD) controls

	**22q11.2DS**	**WS**	**TD**	**Group effect**	
	**n = 55**	**n = 36**	**n = 280**	**df = 2, 368**	**Post Hoc**
**Full-scale IQ**	80.59 (12.60)	67.41 (15.08)		t (89) = 4.1	
				*P* = 0.0001	
**Males/Females**	32/23	14/22	149/131	*P* = 0.18	
**Shyness**	2.65 (0.89)	1.73 (0.63)	2.29 (0.79)	F = 14.6	TD < 22q11.2DS*
				*P* ≤0.0001	WS < 22q11.2DS**
					WS < TD**
**Emotionality**	3.17 (1.19)	3.49 (1.01)	2.74 (0.77)	F = 15.4	TD < 22q11.2DS*
				*P* ≤0.0001	TD < WS**
**Activity**	2.93 (0.96)	3.27 (0.80)	3.70 (0.70)	F = 26.7	WS < TD*
				*P* ≤0.0001	22q11.2DS < TD**
**Sociability**	3.56 (0.76)	3.63 (0.67)	3.64 (0.48)	F = 0.4	
				*P* = 0.58	

Scores are presented as mean (SD).

* *P* <0.01, **P <0.001.

**Figure 1:**
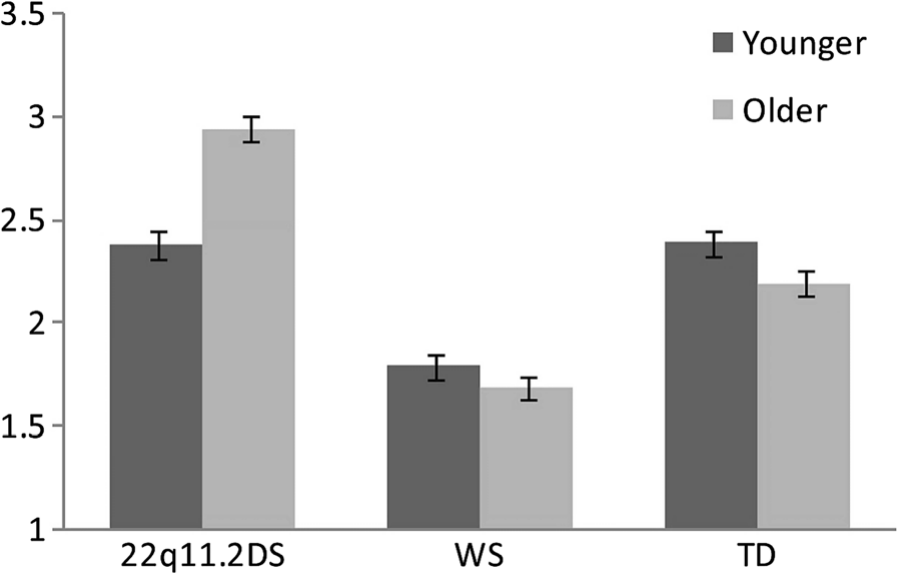
**Comparison of Shyness scores among children with 22q11.2 deletion syndrome (22q11.2DS, n = 55), Williams syndrome (WS, n = 36), and typically developing children (TD, n = 280), as a function of age.** The age difference (below vs. above the median age) is significant in the 22q11.2DS group only. Older children with 22q11.2DS (n = 27) have significantly higher Shyness scores (*P =* 0.02) than younger 22q11.2DS children (n = 28).

In addition to the group main effect on Shyness reported above, there was also a significant group by age interaction on Shyness scores (F(2,368) = 5.7, *P* <0.005). This interaction reflected the fact that older 22q11.2DS individuals had higher shyness scores compared to their younger counterparts (Figure 1). There were no significant group by age interactions in shyness for either WS or TD children (Figure 1). There were also no gender effects on any of the temperament scores. Within the 22q11.2DS and WS samples we found only a weak, although significant, correlation between IQ and shyness (r = 0.23, *P* = 0.05) and no significant correlations between IQ and the other EAS scale scores: Emotionality (r = −0.07, *P* = 0.56), Activity (r = 0.20, *P* = 0.08), and Sociability (r = 0.03, *P* = 0.79). When IQ was entered as a covariate in the analysis with 22q11.2DS vs. WS on each of the EAS scales, all group effects remained the same and no significant IQ effect was noted. Thus, it appears that the differences in shy temperament between 22q11.2DS and WS children are not accounted for by differences in IQ between the groups.

### Temperamental characteristics that predict future 22q11.2DS psychosis

At baseline, none of the 22q11.2DS participants had a psychotic disorder and, at follow-up, 12.2% had a psychotic disorder (3 schizophrenia, 2 psychotic disorder NOS, and 1 psychotic depression). At baseline there were proportionally higher rates of social anxiety disorder in the subgroup of individuals with 22q11.2DS who developed psychosis at follow-up than in the subgroup of individuals who did not (50.0% vs. 11.6%, *P* = 0.02). There were no significant differences among the two subgroups in the rates of other baseline psychiatric disorders: specific phobia (50.0% vs. 23.2%, *P* = 0.16), obsessive compulsive disorder (50.0% vs. 20.9%, *P* = 0.12), ADHD (50.0% vs. 46.5%, *P* = 0.87), ODD (33.3% vs. 18.6%, *P* = 0.40), and dysthymia (16.7% vs. 16.3%, *P* = 0.98).

**Figure 2:**
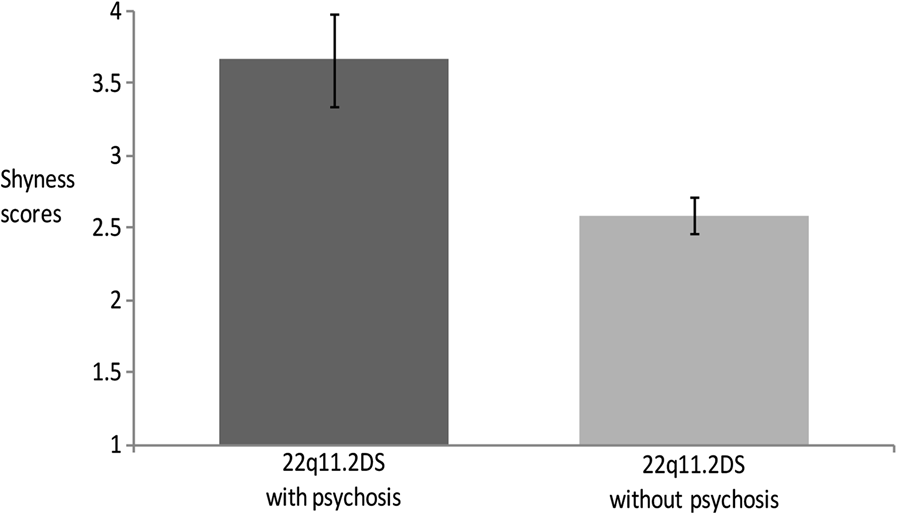
**Comparison of baseline Shyness scores between individuals with 22q11.2DS who developed a psychotic disorder (n = 6) and 22q11.2DS individuals who did not (n = 43).** The 22q11.2DS individuals who developed a psychotic disorder had significantly higher baseline Shyness scores than those who did not (z = −2.7, *P* = 0.007).

To assess if the temperament subscales at baseline predicted the future emergence of a psychotic disorder in 22q11.2DS, we conducted a logistic regression with the presence/absence of a psychotic disorder at follow-up entered as the outcome variable. The predicting variables were gender, age, and full scale IQ at baseline assessment, and the three baseline EAS subscale scores that distinguished between 22q11.2DS and controls (Shyness, Emotionality, and Activity). Of all the potential predictors, only baseline scores of Shyness were significantly associated with the presence of a psychotic disorder at follow-up (Odds Ratio = 1.8, *P* = 0.01), with this regression model accounting for 44% of the variance. The 22q11.2DS individuals who subsequently developed a psychotic disorder had significantly higher baseline Shyness scores than those who did not develop a psychotic disorder (Figure 2).

Although age was not a significant predictor in the logistic regression (and hence did not contribute to the prediction of psychosis), we further wanted to rule out any potential confounding effect of age, in light of our finding (reported above) that shyness was higher among older 22q11.2DS individuals. We therefore conducted an additional analysis in which we compared the shyness scores of the six 22q11.2DS individuals who developed a psychotic disorder to those of an age-matched sub-group (n = 25) of 22q11.2DS individuals who did not develop a psychotic disorder. To match for age, we included in the non-psychotic group only individuals 13 years of age or older at baseline (i.e., the age at baseline of the youngest 22q11.2DS individual who developed psychosis). The 22q11.2DS individuals who developed psychosis (n = 6) were similar to those who did not (n = 24) in baseline age (13.0 ± 2.7 vs. 14.3 ± 3.8, respectively, *P* = 0.46) and follow-up age (17.9 ± 3.8 vs. 19.6 ± 4.5, respectively, *P* = 0.19) but were different in baseline shyness scores, with the psychotic 22q11.2DS individuals having significantly higher baseline shyness scores than the non-psychotic 22q11.2DS individuals (3.7 ± 0.8 vs. 2.6 ± 0.8, respectively, (F(2,47) = −3.0, *P* <0.005).

## Discussion

Our results indicate that children with 22q11.2DS and WS have distinct behavioral phenotypes. Both syndromes show higher Emotionality and lower Activity than TD children. Moreover, consistent with prior findings, children with 22q11.2DS are significantly shyer than children with WS as well as TD controls [33-35]. In addition, age was positively associated with Shyness in the 22q11.2DS group, such that older 22q11.2DS children were perceived by their mothers as shyer than younger 22q11.2DS children. Finally, we found that higher scores on shyness were associated with the subsequent emergence of psychosis in 22q11.2DS.

Children with 22q11.2DS and WS both present with a number of medical comorbidities but we found that children with 22q11.2DS were shyer than children with WS, who were lowest on the shyness measure. The finding is consistent with the known socially disinhibited and outgoing behavior of individuals with WS [33,36,37]. Shyness in children with 22q11.2DS likely has a neural basis (discussed later), but could also partially stem from the numerous physical problems with which these children cope, specifically the palatal abnormalities causing hypernasal speech which is frequently incomprehensible or stands out as having an unusual sound [4]. In contrast, the medical problems of WS children are less physically obvious in nature. Thus, 22q11.2DS children may be more likely to experience ridicule, bullying, or discrimination from peers due to their physical problems, which can in turn promote withdrawn and shy behavior on their part, particularly in older children who are more sensitive to the possibility of social ostracism.

In the present study, we found that 22q11.2DS and WS share temperamental features including high emotionality and low activity compared to TD children. High emotionality is defined as the tendency to become upset easily and intensely. The high emotionality in 22q11.2DS and WS is probably also reflected in the high rates of ADHD and ODD in the two syndromes [5-7]. We believe that the high emotionality of individuals with the two syndromes stems from a biological impairment of mood regulation and disinhibition, and the tendency of these individuals to respond with great intensity when confronted with difficulties [4,38]. Of note, parent–child interactions, particularly around limit-setting, may be more complex and vulnerable in families of children coping with 22q11.2DS and WS compared to families with TD children. For example, parents of a child with 22q11.2DS or WS may rightly feel that greater firmness on their part would lead to more dysregulation in the child, and hence choose to reduce their demands of mature behavior from their child so as not to overly-challenge him or her. However, such a parental approach can provides children with less opportunities to practice social and self-regulatory skills.

Unexpectedly, we did not find differences in Sociability between 22q11.2DS, WS, and TD children. This may have been due to a ceiling effect, as all three groups received quite high ratings on the Sociability subscale, with relatively small standard deviations. Notably, the correlation between Sociability and Shyness scores in our sample was weak, suggesting that they are measuring different constructs (although it is possible that the correlation might have been higher if the Sociability measure had greater variability).

The association between shyness and later onset of a psychotic disorder in 22q11.2DS is a pivotal finding of our study. Because individuals with 22q11.2DS are highly susceptible to developing psychotic disorders, intensive research efforts are invested in finding biomarkers and risk factors for the emergence of psychosis in this population. High levels of anxiety in childhood have been identified as a predictor for the later development of psychosis in 22q11.2DS in a previous study with a different sample [8]. This is also consistent with the current sample, in which individuals with 22q11.2DS who developed psychosis were more likely to have a social anxiety disorder at baseline than those who did not develop psychosis. Moreover, shyness is one of the major manifestations of anxiety and the present findings converge with the previous study [8] to indicate that social inhibition, in particular, is associated with the onset of psychosis in individuals with 22q11.2DS.

There are also a few longitudinal studies in the general population which found that ultra-high-risk prodromal subjects who later undergo the transition to schizophrenia had increased social withdrawal and anhedonia [39,40]. Social incompetence and social withdrawal are related but not identical to shyness, and to our knowledge there are no longitudinal studies in the general population that have investigated shy temperament as a predictor of the later onset of schizophrenia. It should be noted that what is reported as ‘Shyness’ by parents may actually be a prodromal or a negative symptom, because social withdrawal, isolation, and anhedonia that are common in shy individuals are also common manifestations of negative symptoms of schizophrenia and its prodrome [41].

There is accumulating evidence for the biological underpinnings of 22q11.2DS social deficits and their poor face processing abilities [42,43]. A study using fMRI to probe visual recognition in young individuals with 22q11.2DS found abnormal activations in brain areas involved in emotional face processing and in interpretation of social cues in individuals with 22q11.2DS, including anterior superior temporal sulcus, rostral anterior cingulated cortex, and amygdala [44]. The study also found impaired response and adaptation of the amygdala to fearful faces in 22q11.2DS [44]. Another study found a longitudinal decrease in the volume of the mesial temporal lobe of adolescents with 22q11.2DS and the longitudinal volume decrease was associated with an increase in prodromal symptoms in this population [45]. The above imaging findings suggest that children with 22q11.2DS are impaired in basic components of social cognition and emotional regulation such as facial expression processing and adaptation to stressful social situations. Thus, we speculate that the neural impairments of 22q11.2DS individuals, in combination with other factors such as the hypernasal speech and abnormal facial features, lead to a negative self-concept and shy temperament marked by limited expression of affect, limited social engagement, and social withdrawal in novel social situations. It is possible that shyness is an early indicator reflecting neurobiological vulnerability and proneness to psychosis.

The low activity level of 22q11.2DS and WS found in our study is surprising in light of their known high rates of ADHD. Yet, high scores in the activity measure of the EAS may reflect more constructive energetic activity (e.g., playing with peers), which is expressed more in TD children, rather than unconstructive hyperactivity symptoms which are more common in 22q11.2DS and WS.

A notable limitation of the present study is the fact that assessments of temperament were based solely on parental reports, which can of course be prone to bias. Moreover, to our knowledge, the EAS temperament measure has not been previously used in studies with WS or 22q11.2DS individuals. However, this instrument does have several advantages, as it is a well-established and widely used temperament questionnaire, which is suitable for a wide age range (from infancy to young adulthood). Future studies should employ observational measures of temperament, in addition to parental reports, to more thoroughly characterize the temperamental characteristics of individuals with 22q11.2DS and WS. The wide age range of the present sample can be seen as a further weakness, because shy behavior might have different meanings at different ages (especially as perceived by parents). Future studies should examine more homogenous age groups and follow them up longitudinally to discern whether shyness confers greater risk at particular ages.

## Conclusions

In this study, we identified shyness as a characteristic associated with the onset of psychosis in individuals with 22q11.2DS. As shown herein, substantial shyness can be seen quite early in development in 22q11.2DS individuals and is easily identified using a simple parent-report questionnaire. Future studies could indicate whether or not early detection and intervention aimed at decreasing social inhibition and related social avoidance behaviors among individuals with 22q11.2DS can decrease the rate of psychosis in this at-risk population.

## Abbreviations

22q11.2DS: 22q11.2 deletion syndrome; ADHD: Attention deficit/hyperactivity disorder; EAS: Emotionality, activity, sociability temperament survey; ODD: Oppositional defiant disorder; TD: Typically developing; WS: Williams syndrome.

## Competing interests

The authors have no financial or non-financial competing interests to declare.

## Authors’ contributions

YS, MD, AK, AF, AW, and DG designed the study and DG wrote the protocol. AF conducted the molecular diagnoses. YS, MD, AK, HZ, and DG carried out the psychiatric and behavioral evaluations and participated in the analyses of data. YS, HZ, GS, GZ, and DG managed the literature search and statistical analyses. YS wrote the first draft of the manuscript and YS, MD, AK, HZ, GS, GZ, AW, and DG assisted in further preparation of the manuscript. All authors contributed to and have approved the final version of the manuscript.
